# The causal future: The influence of shape features caused by external transformation on visual attention

**DOI:** 10.1167/jov.21.11.17

**Published:** 2021-10-25

**Authors:** Yunyun Chen, Yuying Wang, Sen Guo, Xuemin Zhang, Bihua Yan

**Affiliations:** 1 Beijing Key Laboratory of Applied Experimental Psychology, National Demonstration Center for Experimental Psychology Education, Faculty of Psychology, Beijing Normal University, Beijing, China; 2 Shaanxi Key Laboratory of Behavior and Cognitive Neuroscience, School of Psychology, Shaanxi Normal University, Xi'an, Shaanxi, China; 3 Shaanxi Key Laboratory of Behavior and Cognitive Neuroscience, School of Psychology, Shaanxi Normal University, Xi'an, Shaanxi, China; 4 Beijing Key Laboratory of Applied Experimental Psychology, National Demonstration Center for Experimental Psychology Education, Faculty of Psychology, Beijing Normal University, Beijing, China; 5 State Key Laboratory of Cognitive Neuroscience and Learning and IDG/McGovern Institute for Brain Research, Beijing Normal University, Beijing, China; 6 Shaanxi Key Laboratory of Behavior and Cognitive Neuroscience, School of Psychology, Shaanxi Normal University, Xi'an, Shaanxi, China

**Keywords:** transformation, causal history, shape features, visual attention

## Abstract

Previous studies have validated that participants can distinguish different origins of objects’ shape features, teasing apart features caused by transformation (causal history) from those of the original shape. Considering bite as a transformation example, two experiments were designed to investigate the effect of causal history on the allocation of visual attention. Participants were presented with regular and familiar complete or bitten shapes in Experiment 1 and unfamiliar and irregular complete or bitten shapes in Experiment 2 over a range of stimulus onset asynchronies (SOAs). The task was to identify different probes (i.e., punctuation marks) that equally appeared at four positions around these shapes. The results showed that complete regular shapes had no impact on participants’ reaction times to identify probes that appeared at the four different positions (Experiment 1), whereas complete irregular shapes would facilitate participants’ responses to the probes that appeared at the positions around the “head” of the irregular shape (Experiment 2) regardless of SOAs. When presented with bitten shapes, in the earlier phase of visual processing, participants’ response patterns resembled those found when complete shapes were presented. However, with longer SOAs, participants were faster in identifying probes that appeared at those positions that were around the nontransformed region of the bitten shapes. The results revealed that information about shape features caused by causal history could be incorporated, albeit relatively later, into the allocation of visual attention. The role of causal history in the speculation about one object's future development is discussed.

## Introduction

Shape is one of many important cues for making inferences about object properties (Schmidt & Fleming, 2018). Many tasks, such as object recognition (Landau, Smith, & Jones, 1998), reaching and handling actions (Ansuini, Santello, Tubaldi, Massaccesi, & Castiello, 2007), material perception (Schmidt, Fleming, & Valsecchi, 2020), conceptualization (Green, 2015), and classification (Graf, 2010), are to some extent dependent on objects’ shape features. In the real world, the shape of an object is usually the product of various generative processes, such as manufacture, biological growth, self-organization, and so on. Although these processes can be quite complex, any thing's developments follow certain rules. For example, when eating an apple, the second bite is more likely to be in the same region as the first bite, compared to another random spot on the apple. Therefore, the so-called “shape is time” is precisely because shape transformations are processes based on time. Leyton (1989) proposed the term *causal history*, which refers to the transformations that are applied to an object. The perception of causal history is to infer the process of transformation according to the current shape features. There is evidence showing that observers can distinguish features caused by transformation (causal history) from those that “belong to” the original shape (Schmidt & Fleming, 2018; Schmidt, Phillips, & Fleming, 2019; Schmidt, Spröte, & Fleming, 2016; Spröte & Fleming, 2016).

Leyton (1989) first reported that the inference of causal origin has an important impact on the visual perception of shape and proposed a detailed theoretical framework that solved two problems: inferring the causal history of a single shape and inferring the causal history of the same object in two developmental stages. Individuals can use very simple heuristic rules spontaneously and irresistibly to infer the relationship between the processes that an object experienced. In the study by Spröte, Schmidt, and Fleming (2016), when asked to compute the symmetry axes of figures that were similar in geometry but different in causal origins, individuals seemed to suppress the shape features caused by external forces (bite). The derived symmetry axis was similar to the symmetry axis of the corresponding complete object rather than the axis constrained by the actual geometrical structure. Observers derive different perceptual organizations from shapes with subtle differences in their geometrical structures but significantly different causal origins of their features, showing that individuals can analyze the shapes of objects not only according to what features the objects have but also according to how the objects have these features. Moreover, the perception of causal history has an impact on visual processing. Observers could reconstruct causal history from static shapes (Chen & Scholl, 2016). In addition, in the absence of other clues, the optical appearance of the object will affect the perception of the softness of the object. However, when transformation cues exist, individuals will rely more on transformation than optical cues to infer the softness of objects (Paulun, Schmidt, van Assen, & Fleming, 2017; but see Schmid & Doerschner, 2018; Schmidt, Paulun, Assen, & Fleming, 2017). Taken together, the findings mentioned above demonstrate that the inference of causal history is indeed a general phenomenon in shape perception and shape understanding (Schmidt, Spröte, & Fleming, 2016).

Individuals not only can identify the type of transformation applied to an object but also can determine the region and magnitude of that transformation relatively precisely. Observers can still recognize objects when different transformations are applied to those objects (Fleming & Schmidt, 2019). Moreover, we can also classify objects according to the processes that shape them, regardless of whether or not we have seen them before. These results show that individuals can recognize the shape features caused by causal history as well as the original shape features of objects. On this basis, Schmidt et al. (2019) proposed the “shape scission” hypothesis. Analogous to the scission problem in lightness perception (e.g., Anderson & Winawer, 2005), researchers believe that “shape scission” helps us identify and understand the shape transformations of objects and is potentially important for many perceptual and cognitive tasks, such as promoting object constancy, inferring object materials, and predicting object future behaviors. In a similar vein, Green (2015) proposed a layered view of shape perception, holding that an object is represented as having multiple shape properties with different degrees of abstraction, such as the metric and abstract properties. The former refers to the properties affected by changes in certain distances, lengths, or angles. The latter, which is more stable, refers to the properties that survive similar changes. Therefore, individuals might have established a multidimensional shape feature space when representing objects based on their long-term visual experience and can access different representational layers depending on tasks (Schmidt, Phillips, & Fleming, 2019).

Leyton (1989) believed that we can perceive the past from a single static shape based on the speculation about the generative process that shapes the object and therefore believed that the shape of an object is a window into its past. Even the static objects could be represented in temporal terms, in ways of recapitulating their causal histories (Chen & Scholl, 2016). However, little is known about whether the causal history of an object plays a role in speculation about its future states. There have been some studies focusing on the effect of the object's shape, particularly the original shape, on visual attention and have shed light on this question. For example, Sigurdardottir et al. (2014) demonstrated that information about the directionality of an object can be automatically incorporated into visual orientation and motion estimation because that information often implies where the object will be. Directionality is constrained by the intrinsic features that are constrained by the biological growth of the object. This reveals the role of the original shape features of an object in the speculation about the future state of this object. What about the effect of causal history? The structure and transformation of objects define a unique type of event in space-time (Kim, Effken, & Shaw, 1995). The conjunction of a specific type of change and a specific type of structure dynamically determines a unique trajectory of the continuum of space-time. Change is never random but occurs in repeatable and consistent manners (Kim, Effken, & Shaw, 1995). It is because of this particularity that individuals could retrospectively specify the past based on special shape features. Accordingly, we believe that this predictability and consistency can also make it possible to speculate about the future developments and changes of objects. Observers could already be proficient in the application of this consistency. For example, researchers found that observers did quite well in estimating how points on or near an object shifted in space across a wide range of transformation, including complex nonrigid transformations (Schmidt & Fleming, 2016; Ward, Isik, & Chun, 2018). Therefore, we believe that observers not only can name and classify the objects but also can infer and predict their past and future changes according to the extrinsic shape features caused by transformations. On the other hand, the extrinsic shape features of an object imposed by transformations usually indicate the interaction between the object and other objects or persons. Previous studies about action-related objects have shown that the perceived action between two objects (e.g., a bottle pouring toward a glass) can influence how attention is distributed (Riddoch et al., 2003, 2006), reallocating attention in the direction of an implied action (Roberts & Humphreys, 2011). This effect shows that the most possible changes that could occur in the future bias our attention. Thus, it is hypothesized in this study that extrinsic features could also be incorporated into the allocation of visual attention. Attention precedes action. Based on transformations that have been applied to the object, observers’ attention can be pushed away to regions where transformations might be applied in the future. In a similar vein to Sigurdardottir et al. (2014), we decided to investigate whether causal history influenced attention distribution to investigate our hypothesis, that is, the causal history of an object plays an important role in the speculation about its future changes. Considering the bite as a transformation example, we used familiar and regular shapes in Experiment 1 and focused on the effect of causal history (bite transformation) on visual attention. We also focused on different perceptual organizations derived from different causal origins to investigate the confounding effect of visual complexity of the contours (the lower-level perception of shape features). Furthermore, Experiment 2 adopted irregular shapes with form-derived directionality to verify the robustness of the causal history effect. With answers to these questions, we could further provide evidence for the hypothesis of “shape is time.”

## Experiment 1

Sigurdardottir et al. (2014) found that individuals were faster at detecting visual targets when shapes pointed to their location even though targets were no more likely to appear there than they were to appear in the opposite direction. This result revealed the effect of intrinsic shape features on the allocation of visual attention. The influence on visual attention might result from that the object shape can restrict its movements and therefore its probable future locations since the direction of an object usually implies its possible direction of movement. Accordingly, the extrinsic shape features imply information about the possible force and the force direction. Therefore, we hypothesized that extrinsic shape features can also influence the allocation of visual attention and thus restrict possible future changes.

### Experiment 1a

#### Method

##### Participants

The sample size was determined by a priori power analysis using G∗Power software, version 3.1.9.7 (Faul, Erdfelder, Lang, & Buchner, 2007). The power analysis indicated that at least 24 participants were required to obtain a statistical power of 0.8, assuming a Type I error probability of 0.05 and a medium effect size (*f* = 0.25) for the *F* test on the effect of a four-level within-participant factor (the position, as shown in Design). Twenty-five college students (9 males and 16 females) aged 17–21 years (*M* = 18.47, *SD* = 0.92) with normal or corrected-to-normal vision participated in this study for financial compensation. We recruited a few more participants than required in case that some participants’ data could be deleted. They were all right-handed and naive to the purpose of the experiment. The viewing distance was approximately 80 cm.

##### Stimuli

**Figure 1.**
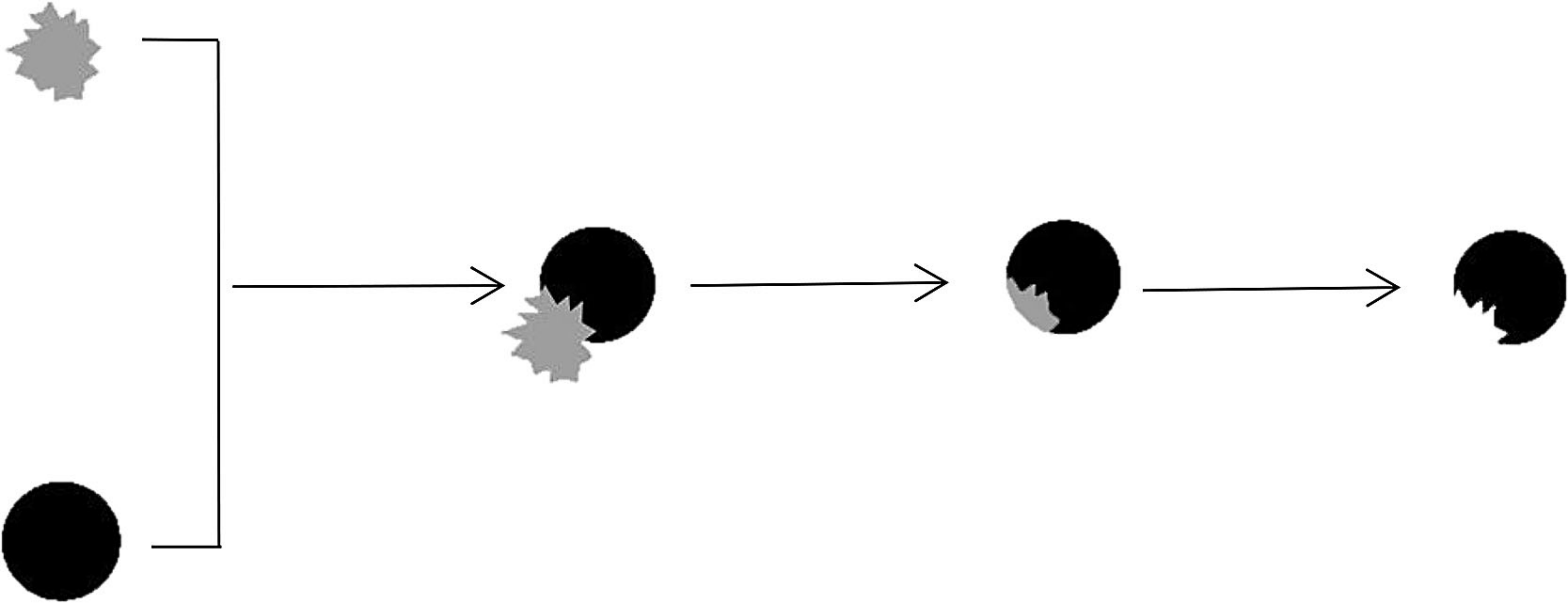
The process of creating a bitten shape is shown.

**Figure 2.**
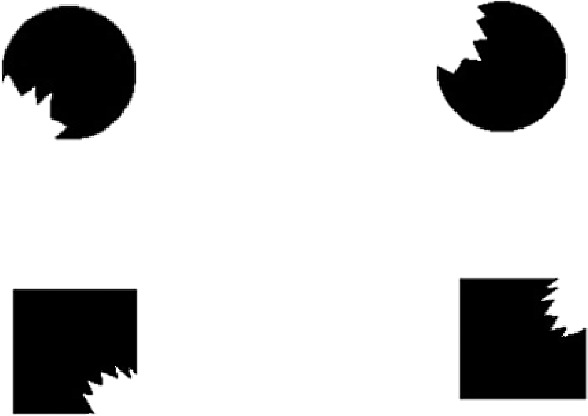
The bitten shapes are shown.

The shapes used in this experiment were all handcrafted with Adobe Photoshop CS6 software (Adobe, San Jose, CA, USA). Some of them were complete ones, and the others (bitten ones) had a part removed by subtracting another shape (see Figure 1). First, we created two black (RGB = 0, 0, 0) base shapes (one circle and one square with a visual angle of 3.22° in diameter) and two different gray (RGB = 192, 192, 192) toothed shapes. Second, we imposed the toothed shape on the base shape and removed the part that extended the base shape. Finally, we changed the color of the gray part to match the background color of the picture, and after rotation to a proper angle, we obtained our target shapes. The jagged outlines of the circles were the same but different from those of the squares (see Figure 2). The area of the part that was removed was approximately 13% of the complete shape.

##### Apparatus

The stimuli were displayed upon (and the data were collected by) a Gateway desktop computer with a refresh rate of 60 Hz and a resolution of 1,024 × 768 pixels (∼18.68° × 14.12°).

##### Procedure

**Figure 3.**
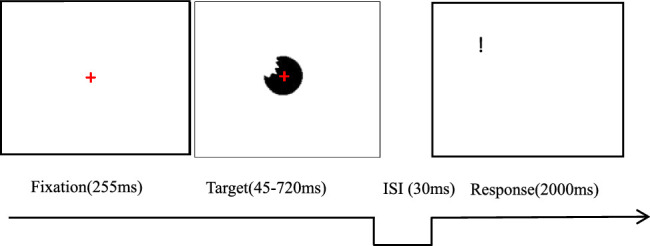
The procedure of Experiment 1a is shown.

As shown in Figure 3, a red cross was displayed in the center of the screen with a white background (RGB = 255, 255, 255) for 255 ms. Then, one object that could be complete or bitten was presented for 45, 105, 225, 420, or 720 ms. Subsequently, the screen went blank for 30 ms. Finally, a punctuation mark that could be a period or exclamation mark appeared randomly at one of four designated positions with equal probability for 2,000 ms. The participants were instructed to press the Z key for the period mark and the “/” key for the exclamation mark within this time. They were asked to fix their eyes on the red cross located at the center of the shape throughout each trial as much as possible.

Considering the center of the screen as the original point, the horizontal and longitudinal distances of the four designated positions from the original point were 20% of the screen length and width, respectively. Therefore, the corresponding relative coordinates were upper left (−204.8, 153.6), upper right (204.8, 153.6), lower left (−204.8, −153.6), and lower right (204.8, −153.6), with the unit of pixels. The participants’ speedy and accurate responses were encouraged.

##### Design

**Figure 4.**
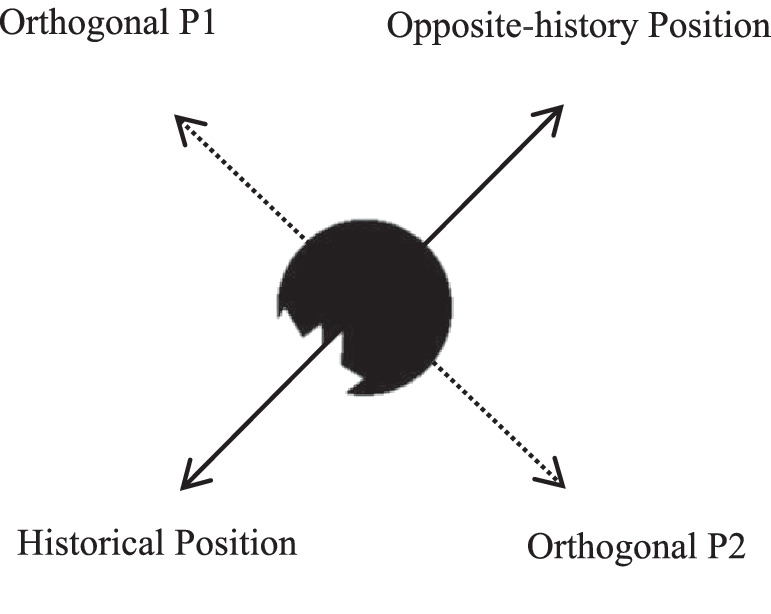
The four positions are shown. The solid line shows the direction of transformation and the dashed line shows the orthogonal direction.

Experiment 1a adopted a within-subject design with 2 targets (complete, bitten) × 4 detection positions (upper left, lower left, upper right, lower left) × 5 SOAs (75, 135, 255, 450, 750 ms) × 2 punctuation marks (period, exclamation) × 4 replications. The trials were presented in a different random order for each participant. As shown in Figure 4, the four positions around the bitten shape were divided into two groups: transformation positions and orthogonal positions. The transformation group had two positions, defined as historical and opposite-history positions, respectively. The historical position was so called because it was near the area where the transformation had occurred. The opposite-history position was defined just because it was opposite the historical position and had no specific meaning. The other two positions were included in the orthogonal group. Note that the four positions around the bitten shape would be renamed according to where the concavity faced. The four positions around the complete shape would be referred to as upper left, upper right, lower left, and lower right positions.

#### Results

Incorrect trials (3.7% of trials) were deleted. Trials in which reaction time (RT) was less than 150 ms and greater or less than 3 *SD* from an individual's mean RT (1.2% of trials) were eliminated from further analysis.

##### Data of complete shapes

**Figure 5.**
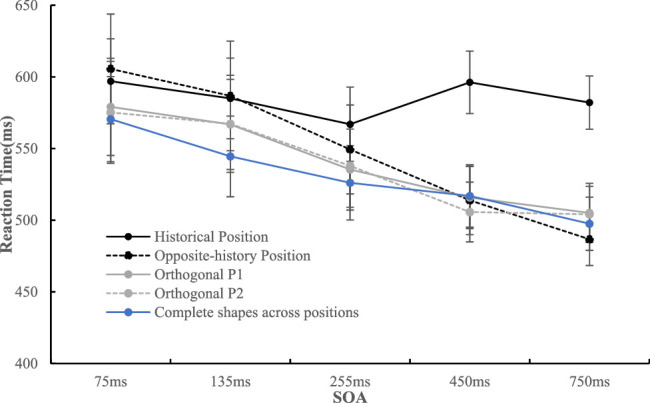
The RT mean under each condition is shown. The error bar represents standard error.

This part of the data (Figure 5) was analyzed in a 4 (position: upper left, upper right, lower left, lower right) × 5 (SOA: 75, 135, 255, 450, 750 ms) repeated-measures analysis of variance (ANOVA). Greenhouse–Geisser correction was applied when the sphericity assumption was violated. The effect size *d* was calculated by dividing the mean of the differences between conditions by their standard deviation (Cohen's *d_z*). The results showed that only the main effect of SOA was significant, *F*(2.51, 60.18) = 9.85, *p* < 0.001, η_p_^2^ = 0.291, and Bonferroni-corrected pairwise comparisons revealed that participants were faster to identify the probe when the SOA was 75 ms (*M* = 570.52 ms, *SD* = 148.23) compared to 255 ms (*M* = 526.10 ms, *SD* = 129.54, *t*(24) = 4.12, *p* = 0.004, *d_z* = 0.82), 450 ms (*M* = 516.95 ms, *SD* = 108.97, *t*(24) = 4.23, *p* = 0.003, *d_z* = 0.84), and 750 ms (*M* = 497.54 ms, *SD* = 92.89, *t*(24) = 4.48, *p* = 0.002, *d_z* = 0.90), except for 135 ms (*M* = 544.53 ms, *SD* = 140.65). The results indicated that complete circle or square shapes do not have a significant influence on the allocation of visual attention.

##### Data of bitten shapes

This part of the data (Figure 5) was also analyzed in a 4 (position: historical position, opposite-history position, orthogonal P1, orthogonal P2) × 5 (SOA: 75, 135, 255, 450, 750 ms). The results showed that the main effect of position was significant, *F*(1.50, 36.10) = 12.45, *p* < 0.001, η_p_^2^ = 0.342, and post hoc comparisons with Bonferroni's correction revealed a longer RT mean with the historical position (*M* = 585.47 ms, *SD* = 180.41) than with the opposite-history position (*M* = 548.47 ms, *SD* = 143.49, *t*(24) = 4.12, *p* = 0.002, *d_z* = 0.82), the orthogonal P1 (*M* = 540.47 ms, *SD* =130.42, *t*(24) = 3.62, *p* = 0.008, *d_z* = 0.72), and orthogonal P2 (*M* = 538.06 ms, *SD* = 133.63, *t*(24) = 4.10, *p* = 0.002, *d_z* = 0.82). The main effect of SOA was also significant, *F*(1.32, 31.64) = 15.43, *p* < 0.001, η_p_^2^ = 0.391, and post hoc comparisons with Bonferroni's correction revealed a longer RT with 75 ms (*M* = 589.23 ms, *SD* = 177.80) than 255 ms (*M* = 547.43 ms, *SD* = 149.55, *t*(24) = 5.62, *p* < 0.001, *d_z* = 1.12), 450 ms (*M* = 532.93 ms, *SD* = 127.19, *t*(24) = 4.47, *p* = 0.002, *d_z* = 0.89), and 750 ms (*M* = 519.52 ms, *SD* = 110.93, *t*(24) = 4.22, *p* = 0.003, *d_z* = 0.84), except for 135 ms (*M* = 576.47 ms, *SD* = 171.57). In addition, longer RTs of 135 ms than 255 ms (*t*(24) = 4.56, *p* = 0.001, *d_z* = 0.91), 450 ms (*t*(24) = 4.02, *p* = 0.005, *d_z* = 0.80), and 750 ms (*t*(24) = 3.69, *p* = 0.012, *d_z* = 0.74) were also found. These effects were qualified by a significant interaction, *F*(3.73, 89.44) = 5.41, *p* = 0.001, η_p_^2^ = 0.184. Simple effect analysis showed that there was no significant effect of position when the SOAs were 75 (*F*(3, 22) = 2.29, *p* > 0.10), 135 (*F*(3, 22) = 1.36, *p* > 0.28), and 255 ms (*F*(3, 22) = 2.64, *p* > 0.07). When the SOA was 450 ms, the effect of position was significant, *F*(3, 22) = 4.18, *p* = 0.018, η_p_^2^ = 0.363, and the historical position (*M* = 596.25 ms, *SD* = 199.97) resulted in a longer RT mean than the opposite-history position (*M* = 513.89 ms, *SD* = 119.47, *t*(24) = 3.57, *p* = 0.009, *d_z* = 0.71), orthogonal P1 (*M* = 515.86 ms, *SD* = 108.38, *t*(24) = 3.56, *p* = 0.009, *d_z* = 0.71), and orthogonal P2 (*M* = 505.73 ms, *SD* = 104.20, *t*(24) = 3.64, *p* = 0.008, *d_z* = 0.73). When the SOA was 750 ms, the effect of position was also significant, *F*(3, 22) = 5.40, *p* = 0.006, η_p_^2^ = 0.424. The historical position (*M* = 582.11 ms, *SD* = 184.85) resulted in a longer RT than the opposite-history position (*M* = 486.70 ms, *SD* = 91.61, *t*(24) = 4.00, *p* = 0.003, *d_z* = 0.80), orthogonal P1 (*M* = 505.13 ms, *SD* = 102.99, *t*(24) = 3.03, *p* = 0.035, *d_z* = 0.61), and orthogonal P2 (*M* = 504.13 ms, *SD* = 97.61, *t*(24) = 3.19, *p* = 0.002, *d_z* = 0.64).

### Experiment 1b

The purpose of Experiment 1b was to investigate whether the findings of Experiment 1a resulted from the influence of expectation. The positions in Experiment 1a were tested in one block; thus, the participants could group the opposite-history position and orthogonal P1 and P2 together because they were near the nontransformed regions of the object. Therefore, the participants could identify probes that appeared at these positions more quickly compared to the historical position.

#### Method

##### Participants

Twenty-six college students (12 males and 14 females) aged 19–27 years (*M* = 24.04, *SD* = 2.03) with normal or corrected-to-normal vision participated in this study for financial compensation. They were all right-handed and naive to the purpose of this experiment. The sample size was calculated as in Experiment 1a.

##### Apparatus, stimuli, procedure, and design

The apparatus, stimuli, procedure, and design were the same as in Experiment 1a with the following exception: The four positions were tested in two blocks to investigate the effect of expectation. In one block, the punctuation mark would appear at the historical or opposite-history position. In the other block, the punctuation mark would appear at orthogonal P1 or P2. The trials with complete shapes in the two blocks were the same.

#### Results

Incorrect trials (3.81% of trials) were deleted. Trials in which reaction time was less than 150 ms and greater or less than 3 *SD* from an individual's mean reaction time (1.16% of trials) were eliminated from further analysis.

##### Data of complete shapes

**Figure 6.**
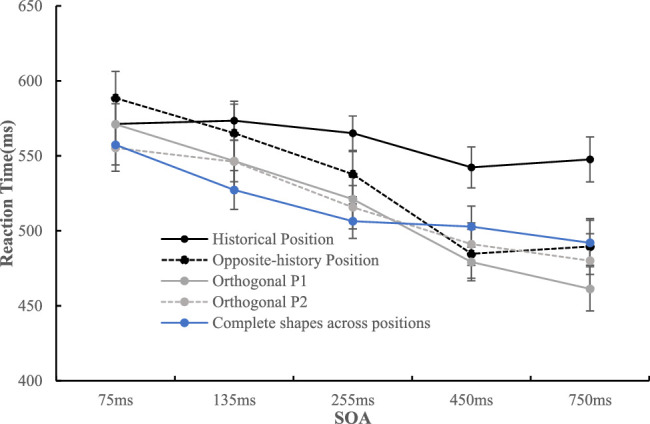
The RT mean under each condition is shown. The error bar represents standard error.

This part of the data (Figure 6) was analyzed in a 4 (position: upper left, upper right, lower left, lower right) × 5 (SOA: 75, 135, 255, 450, 750 ms) repeated-measures ANOVA. The results showed that only the main effect of the SOA was significant, *F*(1.57, 39.35) = 10.72, *p* = 0.001, η_p_^2^ = 0.300, and post hoc comparisons with Bonferroni's correction revealed that 75 ms (*M* = 557.37 ms, *SD* = 67.26) resulted in a longer RT mean than 135 ms (*M* = 527.25 ms, *SD* = 64.76, *t*(25) = 6.39, *p* < 0.001, *d_z* = 1.25), 255 ms (*M* = 506.43 ms, *SD* = 57.40, *t*(25) = 6.20, *p* < 0.001, *d_z* = 1.22), 450 ms (*M* = 502.80 ms, *SD* = 68.26, *t*(25) = 4.24, *p* = 0.003, *d_z* = 0.83), and 750 ms (*M* = 492.01 ms, *SD* = 75.18, *t*(25) = 4.19, *p* = 0.003, *d_z* = 0.82).

##### Data of bitten shapes

This part of the data (Figure 6) was also analyzed in a 4 (position: historical position, opposite-history position, orthogonal P1, orthogonal P2) × 5 (SOA: 75, 135, 255, 450, 750 ms) repeated-measures ANOVA. The results showed that the main effect of position was significant, *F*(1.91, 47.63) = 10.03, *p* < 0.001, η_p_^2^ = 0.286, and post hoc comparisons with Bonferroni's correction revealed a longer RT mean with the historical position (*M* = 559.97 ms, *SD* = 82.43) than with the opposite-history position (*M* = 533.14 ms, *SD* = 68.04, *t*(25) = 3.14, *p* = 0.025, *d_z* = 0.62), the orthogonal P1 (*M* = 515.84 ms, *SD* = 50.78, *t*(25) = 3.97, *p* = 0.003, *d_z* = 0.78), and orthogonal P2 (*M* = 517.64 ms, *SD* = 59.39, *t*(25) = 3.61, *p* = 0.008, *d_z* = 0.71). The main effect of the SOA was also significant, *F*(1.37, 34.12) = 15.52, *p* < 0.001, η_p_^2^ = 0.383. Post hoc comparisons with Bonferroni's correction revealed that 75 ms (*M* = 571.53 ms, *SD* = 79.05) resulted in a longer RT than 255 ms (*M* = 534.93 ms, *SD* = 68.15, *t*(25) = 5.32, *p* < 0.001, *d_z* = 1.04), 450 ms (*M* = 499.32 ms, *SD* = 62.40, *t*(25) = 4.41, *p* = 0.002, *d_z* = 0.87), and 750 ms (*M* = 494.64 ms, *SD* = 68.88, *t*(25) = 4.35, *p* = 0.002, *d_z* = 0.85), except for 135 ms (*M* = 557.82 ms, *SD* = 78.78). In addition, 135 ms resulted in a longer RT mean than 255 ms (*t*(25) = 3.34, *p* = 0.026, *d_z* = 0.65), 450 ms (*t*(25) = 4.04, *p* = 0.004, *d_z* = 0.79), and 750 ms (*t*(25) = 3.90, *p* = 0.006, *d_z* = 0.76), and 255 ms resulted in a longer RT mean than 450 ms (*t*(25) = 3.12, *p* = 0.044, *d_z* = 0.61) and 750 ms (*t*(25) = 3.13, *p* = 0.046, *d_z* = 0.61). These effects were qualified by a significant interaction, *F*(4.76, 118.91) = 4.00, *p* < 0.001, η_p_^2^ = 0.138. Simple effect analysis showed that there was no significant difference among these positions when the SOAs were 75 ms (*F*(3, 23) = 3.20, *p* = 0.042, η_p_^2^ = 0.294^1^) and 135 ms (*F*(3, 23) = 1.23, *p* > 0.32). The effect of position was significant when the SOA was 255 ms, *F*(3, 23) = 3.84, *p* = 0.023, η_p_^2^ = 0.334, and the historical position (*M* = 565.13 ms, *SD* = 88.93) resulted in a longer RT than the orthogonal P1 (*M* = 521.11 ms, *SD* = 73.46, *t*(25) = 3.12, *p* = 0.027, *d_z* = 0.61) and orthogonal P2 (*M* = 515.70 ms, *SD* = 72.20, *t*(25) = 3.53, *p* = 0.015, *d_z* = 0.66). When the SOA was 450 ms, the effect of position was also significant, *F*(3, 23) = 4.79, *p* = 0.010, η_p_^2^ = 0.384, and the participants were slowest in recognizing the marks that appeared at the historical position (*M* = 542.32 ms, *SD* = 87.19) than at the opposite-history position (*M* = 484.65 ms, *SD* = 81.34, *t*(25) = 3.45, *p* = 0.012, *d_z* = 0.68), orthogonal P1 (*M* = 479.21 ms, *SD* = 62.78, *t*(25) = 3.56, *p* = 0.009, *d_z* = 0.70), and orthogonal P2 (*M* = 491.11 ms, *SD* =70.70, *t*(25) = 2.87, *p* = 0.049, *d_z* = 0.56). The effect of position was significant when SOA was 750 ms, *F*(3, 23) = 7.36, *p* = 0.001, η_p_^2^ = 0.490. The participants were slowest in recognizing the marks that appeared at the historical position (*M* = 547.64, *SD* = 83.26) than at the opposite-history position (*M* = 489.56 ms, *SD* = 93.07, *t*(25) = 2.96, *p* = 0.040, *d_z* = 0.58), orthogonal P1 (*M* = 461.29 ms, *SD* = 73.38, *t*(25) = 4.44, *p* = 0.001, *d_z* = 0.87), and orthogonal P2 (*M* = 480.05 ms, *SD* = 90.13, *t*(25) = 3.17, *p* = 0.024, *d_z* = 0.62).

### Discussion

On the one hand, the analysis of complete shapes showed that participants identified targets that appeared at four positions equally rapidly when they were presented with complete circles or squares regardless of SOAs. This outcome might have occurred because both circles and squares are axisymmetric and have multiple axes of symmetry. These shapes do not have any apparent directionality and thus do not have a significant effect on the allocation of visual attention. On the other hand, when the SOA became longer, the participants were faster in identifying probes that appeared at positions near the nontransformed region of the bitten shape. This might have occurred because, with a longer SOA, the participants had more time to process the shape contour and derive a bitten shape representation, leading to paying more attention to the still-existing area. Since the transformation is physically irreversible, paying more attention to the nontransformed region can help avoid missing the possible changes in the future. Due to the limited information provided by a single static shape, future changes are hard to be accurately predicted. Additionally, the task in the current experiment was not demanding. That might be the reason why attention is paid to all parts of the shape on which future transformation could be applied. Another possible explanation is the expectation effect. Compared with the historical position that is near the bitten part, the other three positions can be regarded as near the none-bitten part. Therefore, the probability of the mark appearing near the none-bitten part is relatively greater. When the processing time is longer, individuals have more time to process the details, rendering the distinction more obvious and facilitating the responses to the marks that appeared near the none-bitten part. However, after we controlled for the expectation effect (Experiment 1b), we obtained similar results as in Experiment 1a.

In other words, the results of Experiments 1a and 1b show that causal history in objects can affect spatial visual attention. With a longer processing time, information about causal history can draw spatial visual attention to anywhere where transformation might be applied in the future. However, the part of the shape with the bite removed is always the part of the shape with more visual complexity. More complex stimuli could drive slower response times. Although it is less likely the reason for the results of Experiment 1 because the orthogonal positions were also near the complex (bitten) part, we still investigated this possibility in Experiment 1c.

### Experiment 1c

#### Method

##### Participants

Twenty-five college students (4 males and 21 females) aged 19–25 years (*M* = 21.48, *SD* = 2.29) with normal or corrected-to-normal vision participated in this study for financial compensation. They were all right-handed and naive to the purpose of this experiment. The sample size was calculated as in Experiment 1a.

##### Apparatus, stimuli, procedure, and design

**Figure 7.**
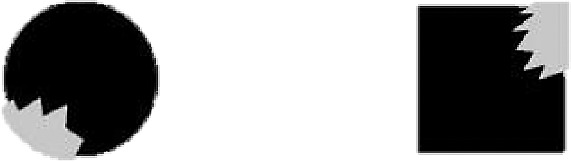
The new shape examples used in Experiment 1c are shown.

The apparatus, stimuli, procedure, and design were the same as in Experiment 1b with the following exceptions. First, the shapes were presented for 420 ms only. This SOA was when the effect of causal history on visual attention was observed. Second, we created new shapes (see Figure 7). However, we did not change the toothed shape's color (RGB = 192, 192, 192). In addition, we translated the gray part a little bit beyond the black shape to ensure that this imposed shape shared a similar outline as the bitten shape but resulted in an apparently different perceptual organization. Third, at the end of the experiment, the participants were first asked to categorize the shapes into complete, bitten, and imposed shapes. After they categorized the shapes, they needed to provide ratings on a scale of 1 to 7 about how representative they thought the shapes were of that type of shape. The higher the number is, the more representative that the participants thought the shapes were. Each participant received 192 trials with 3 targets (complete, bitten, imposed) × 4 detection positions (upper left, lower left, upper right, lower left) × 2 punctuation marks (period, exclamation) × 8 replications.

#### Results

Incorrect trials (3.85% of total trials) were deleted. Trials in which reaction time was less than 150 ms and greater or less than *3* SD from an individual's mean reaction time (1.06% of total trials) were eliminated from further analysis. Categorization task results showed that the causal origins of shapes were perceived as we intended: Complete shapes were undoubtedly perceived as complete (*M* = 7.00, *SD* = 0), bitten shapes were also perceived as bitten (*M* = 6.00, *SD* = 1.56), and imposed shapes were perceived as imposed (*M* = 4.84, *SD* = 1.75). The rating data were submitted to a one-way ANOVA with shape type on three levels (complete, bitten, impose), and the results revealed a significant main effect of shape type, *F*(2, 74) = 18.57, *p* < 0.001. Multiple comparisons with Bonferroni's correction showed that the ratings of complete shapes were higher than that of bitten and imposed shapes (*p*s < 0.001), but the rating of bitten and imposed shapes did not differ between each other. Therefore, the participants interpreted similar jagged outlines according to different causal origins.

##### Data of complete shapes

This part of the data was analyzed in a one-way ANOVA with position on four levels (upper left, upper right, lower left, lower right). The main effect was not significant, *F*(3, 96) = 0.34, *p* = 0.80.

##### Data of bitten and imposed shapes

**Figure 8.**
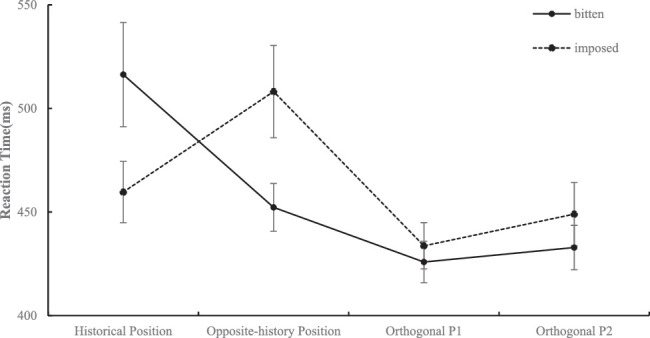
The reaction time under each condition when the target is the bitten shape or imposed shape is shown.

This part of the data (Figure 8) was also analyzed in a 4 (position: historical position, opposite-history position, orthogonal P1, orthogonal P2) × 2 (shape: bitten, imposed) repeated-measures ANOVA. The results showed that the main effect of shape did not reach significance, *F*(1, 24) = 2.18, *p* > 0.15. The main effect of position was significant, *F*(1.35, 32.56) = 9.90, *p* = 0.002, η_p_^2^ = 0.292, qualified by a significant interaction effect of shape and position, *F*(1.51, 36.22) = 11.42, *p* < 0.001, η_p_^2^ = 0.322. Simple effect analysis with Bonferroni's correction showed that when the participants were presented with bitten shapes, they were slower to identify the probe that appeared at the historical position (*M* = 516.34 ms, *SD* = 125.99) than that at the opposite-history position (*M* = 452.25 ms, *SD* = 57.62, *t*(24) = 3.27, *p* = 0.019, *d_z* = 0.65), orthogonal P1 (*M* = 425.89 ms, *SD* = 50.12, *t*(24) = 3.92, *p* = 0.004, *d_z* = 0.78), and orthogonal P2 (*M* = 432.85 ms, *SD* = 53.61, *t*(24) = 3.65, *p* = 0.008, *d_z* = 0.73). In addition, the probe that appeared at orthogonal P1 was identified significantly faster than that at the opposite-history position, *t*(24) = 3.24, *p* = 0.021, *d_z* = 0.65. When the participants were presented with imposed shapes, they were significantly slower to identify the probe that appeared at the opposite-history position (*M* = 508.13 ms, *SD* = 111.27) than that at the historical position (*M* = 459.61 ms, *SD* = 74.05, *t*(24) = 3.67, *p* = 0.007, *d_z* = 0.73) and orthogonal P1 (*M* = 433.69 ms, *SD* = 55.77, *t*(24) = 3.83, *p* = 0.005, *d_z* = 0.77) and were slightly slower than that at orthogonal P2 (*M* = 448.99 ms, *SD* = 76.33, *p* = 0.074).

#### Discussion

Experiment 1c used imposed shapes that shared similar outlines with bitten shapes to investigate the effect of the visual complexity of jagged outlines on visual attention. Interestingly, the participants were faster in identifying the positions around the complex part of the imposed shape. This result was different from the response pattern to the bitten shape. The difference between the two types of shapes could arise from different perceptual organizations. Different shape features provide different information about the generative processes of these features. The features of bitten shapes were caused by transformations and might result in a sense of loss, which led to a “prospection bias,” as we found in previous experiments. The effect of imposed shape could be related to amodal completion, meaning that our visual system ultimately represents partly occluded objects as completed forms (Guttman, Sekuler, & Kellman, 2003), and this process can be completed only in 100–200 ms (Sekuler & Palmer, 1992). After the imposed shape being amodally completed, the jagged outline served as a salient feature and facilitated our responses. Therefore, visual complexity cannot explain the causal history effect.

## Experiment 2

In this experiment, we further explore the different effects of directionality derived from the object's intrinsic shape features and causal history inferred from the object's extrinsic shape features on visual attention. The directionality bias is a well-demonstrated effect, and we want to explore whether causal history can disrupt this bias. If so, we will verify the robustness of the causal history effect. Since the visual complexity cannot account for the slower RT effect at the historical position, Experiment 2 focused on the complete and bitten shapes.

### Experiment 2a

#### Method

##### Participants

Twenty-six college students (10 males and 16 females) aged 18–23 years (*M* = 19, *SD* = 1.26) with normal or corrected-to-normal vision participated in this study for financial compensation. They were all right-handed and naive to the purpose of this experiment. The sample size was calculated as in Experiment 1a.

##### Stimuli

**Figure 9.**
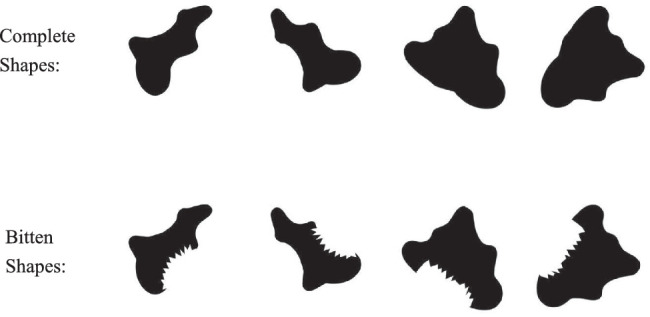
The four complete shapes (the upper row) and four bitten shapes (the lower row) used in Experiment 2 are shown.

The bitten shapes were eight solid black (RGB = 0, 0, 0) novel shapes produced from four nontransformed shapes with different orientations (as shown in Figure 9). The complete shapes were selected and adapted from a previous study (Spröte, Schmidt, & Fleming, 2016). The bitten shapes had a part removed by subtracting another shape, as in Experiment 1. The area of the removed part was about 8% of Shape 1 and 15% of Shape 2. The complete shapes were approximately 5.51∼6.43° in visual angle.

##### Apparatus, procedure, and design

**Figure 10.**
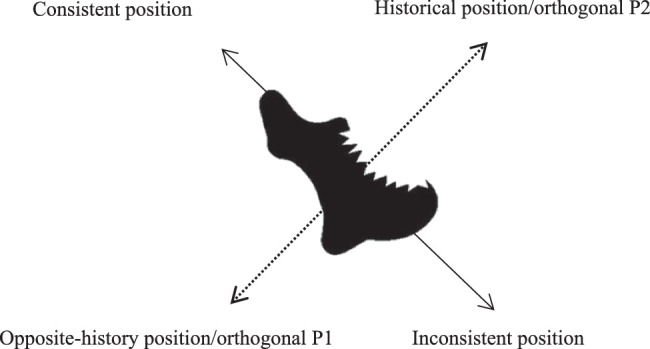
The positions are shown. The solid line shows the inherent direction of the shape and the dashed line shows the orthogonal direction (when the shape is complete) or transformation direction (when the shape is bitten).

The apparatus, procedure, and design were the same as in Experiment 1a with the following exceptions. First, before the formal experiment, the participants were asked to judge the direction of each shape (e.g., whether the shape pointed somewhere and where the shape pointed). Second, according to the original shape features, the two orthogonal positions were referred to as the consistent position near to the “head” of the shape and the inconsistent position opposite the “head” (see Figure 10).

#### Results

A total of 92.3% (24 of 26) of the participants believed that the shapes used in Experiment 2a pointed somewhere, and 91.7% (22 of 24) of these participants thought that the shapes pointed where the smaller end of the shapes appeared to be heading.

Incorrect trials (3.83% of trials) were deleted. Trials in which reaction time was less than 150 ms and greater or less than 3 *SD* from an individual's mean reaction time (1.36% of trials) were eliminated from further analysis.

##### Data of complete shape

**Figure 11.**
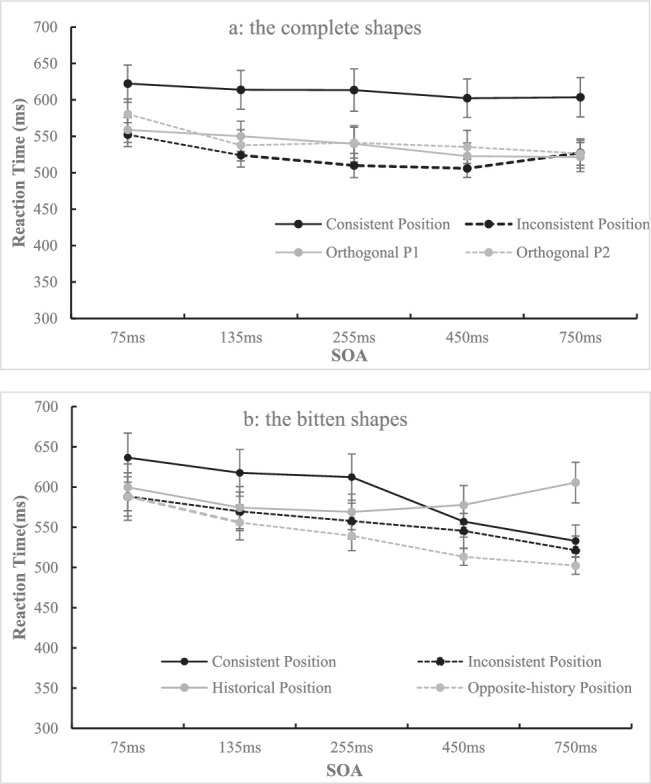
The reaction time under each condition when the target is the complete shape (a) or the bitten shape (b) is shown. The error bar represents standard error.

This part of the data (see Figure 11a) was analyzed in a 4 (position: consistent position, inconsistent position, orthogonal P1, orthogonal P2) × 5 (SOA: 75, 135, 255, 450, 750 ms) repeated-measures ANOVA. The results showed that the main effect of position was significant, *F*(1.19, 29.84) = 10.82, *p* < 0.001, η_p_^2^ = 0.302, and post hoc comparisons with Bonferroni's correction revealed that the consistent position (*M* = 611.11 ms, *SD* = 134.94) resulted in a longer RT mean than the inconsistent position (*M* = 523.91 ms, *SD* = 74.24, *t*(25) = 3.75, *p* = 0.006, *d_z* = 0.73), orthogonal P1 (*M* = 538.56 ms, *SD* = 97.93, *t*(25) = 3.20, *p =* 0.022, *d_z* = 0.63), and orthogonal P2 (*M* = 544.26 ms, *SD* = 104.07, *t*(25) = 3.00, *p* = 0.036, *d_z* = 0.59). The main effect of the SOA was also significant, *F*(4, 100) = 13.79, *p* < 0.001, η_p_^2^ = 0.356, and post hoc comparisons with Bonferroni's correction revealed that 75 ms (*M* = 578.50 ms, *SD* = 90.89) resulted in a longer RT mean than 135 ms (*M* = 556.41 ms, *SD* = 97.50, *t*(25) = 4.40, *p* = 0.002, *d_z* = 0.86), 255 ms (*M* = 551.07 ms, *SD* = 101.59, *t*(25) = 4.40, *p* = 0.002, *d_z* = 0.86), 450 ms (*M* = 541.62 ms, *SD* = 83.48, *t*(25) = 6.80, *p* < 0.001, *d_z* = 1.33), and 750 ms (*M* = 544.71 ms, *SD* = 90.60, *t*(25) = 5.58, *p* < 0.001, *d_z* = 1.09). These effects were qualified by a slightly significant interaction, *F*(12, 300) = 2.12, *p* = 0.015, η_p_^2^ = 0.078. Simple analysis showed that, when the SOAs were 75 ms and 450 ms, the difference between the consistent and orthogonal P2 did not reach significance, which was different from what was revealed by the main effect of position.

##### Data of bitten shape

This part of the data (see Figure 11b) was analyzed in a 4 (position: consistent position, inconsistent position, historical position, opposite-history position) × 5 (SOA: 75, 135, 255, 450, 750 ms) repeated-measures ANOVA. The results showed that the main effect of position was significant, *F*(1.59, 39.84) = 8.62, *p* = 0.002, η_p_^2^ = 0.256, and post hoc comparisons with Bonferroni's correction showed that the opposite-history position (*M* = 539.84 ms, *SD* = 80.27) resulted in a shorter RT than the consistent position (*M* = 591.36 ms, *SD* = 116.56, *t*(25) = 3.62, *p* = 0.008, *d_z* = 0.71) and historical position (*M* = 585.28 ms, *SD* = 113.94, *t*(25) = 3.60, *p* = 0.008, *d_z* = 0.71). The main effect of the SOA was also significant, *F* (1.56, 39.06) = 14.70, *p* < 0.001, η_p_^2^ = 0.370, and post hoc comparisons with Bonferroni's correction showed that 75 ms (*M* = 603.23 ms, *SD* = 132.38) resulted in a longer RT than 135 ms (*M* = 579.46 ms, *SD* = 113.92, *t*(25) = 4.10, *p* = 0.004, *d_z* = 0.80), 255 ms (*M* = 569.65 ms, *SD* = 102.02, *t*(25) = 3.86, *p* = 0.007, *d_z* = 0.76), 450 ms (*M* = 548.41 ms, *SD* = 81.86, *t*(25) = 4.51, *p* = 0.001, *d_z* = 0.88), and 750 ms (*M* = 540.58 ms, *SD* = 72.42, *t*(25) = 4.27, *p* = 0.002, *d_z* = 0.84). In addition, 135 ms resulted in a longer RT than 450 ms (*t*(25) = 3.56, *p* = 0.015, *d_z* = 0.70) and 750 ms (*t*(25) = 3.47, *p* = 0.019, *d_z* = 0.68), and 255 ms resulted in a longer RT than 450 ms (*t*(25) = 3.25, *p* = 0.033, *d_z* = 0.64) and 750 ms (*t*(25) = 3.71, *p* = 0.010, *d_z* = 0.73). These effects were qualified by a significant interaction, *F*(3.10, 77.55) = 4.35, *p* = 0.006, η_p_^2^ = 0.148. Simple analysis revealed that there was no significant effect of position on RT when SOAs were 75 ms (*F*(3, 23) = 1.90, *p* > 0.15) and 135 ms (*F*(3, 23) = 2.51, *p* > 0.08). Position had a significant effect on RT when the SOA was 255 ms, *F*(3, 23) = 4.90, *p* = 0.009, η_p_^2^ = 0.390, and the opposite-history position (*M* = 539.45 ms, *SD* = 92.91) resulted in a shorter RT than the consistent position (*M* = 612.36 ms, *SD* = 143.53, *t*(25) = 3.21, *p* = 0.022, *d_z* = 0.63). When the SOA was 450 ms, the effect of position on RT was significant, *F*(3, 23) = 4.42, *p* = 0.014, η_p_^2^ = 0.366, and the opposite-history position (*M* = 513.27 ms, *SD* = 53.05) resulted in a shorter RT than the consistent position (*M* = 557.07 ms, *SD* = 97.07, *t*(25) = 3.43, *p* = 0.013, *d_z* = 0.67) and historical position (*M* = 577.69 ms, *SD* = 120.57, *t*(25) = 3.06, *p* = 0.031, *d_z* = 0.60). The effect of position was also significant when the SOA was 750 ms, *F*(3, 23) = 5.63, *p* = 0.005, η_p_^2^ = 0.423. The historical position (*M* = 605.57 ms, *SD* = 126.33) resulted in a longer RT than the consistent position (*M* = 533.01 ms, *SD* = 99.05, *t*(25) = 3.04, *p* = 0.033, *d_z* = 0.60), the inconsistent position (*M* = 521.47 ms, *SD* = 87.61, *t*(25) = 3.68, *p* = 0.007, *d_z* = 0.72), and the opposite-history position (*M* = 502.28 ms, *SD* = 52.93, *t*(25) = 4.21, *p* = 0.002, *d_z* = 0.83).

### Experiment 2b

Experiment 2b also aimed to investigate the effect of expectation on the results of Experiment 2a. When presented with bitten shapes, the participants might group the opposite-history, consistent, and inconsistent positions together because these positions were close to the part of the object that remained intact. When presented with complete shapes, the participants might group the inconsistent position, orthogonal P1, and orthogonal P2 together because these positions were all far from the head of the shape. Such possibility might also be the reason why the participants identified the probes that appeared at the historical position or the consistent position more slowly compared to other positions, especially when the shapes were presented for a longer time.

#### Method

##### Participants

Twenty-five college students (6 males and 19 females) aged 20–27 years (*M* = 23.64, *SD* = 2.34) with normal or corrected-to-normal vision participated in this study for financial compensation. They were all right-handed and naive to the purpose of this experiment. The sample size was calculated as in Experiment 1a.

##### Apparatus, stimuli, procedure, and design

The apparatus, stimuli, procedure, and design were the same as in Experiment 2a with the following exception: The four positions were tested in two blocks to investigate the effect of expectation. In one block, the punctuation mark would appear at the historical or opposite-history position. In the other block, the punctuation mark would appear at the consistent or inconsistent position.

#### Results

Ninety-two percent (23 of 25) of the participants believed that the shapes used in Experiment 2b pointed somewhere, and 100% (23 of 23) of the participants thought that the shapes pointed where the smaller end of the shapes appeared to be heading.

Incorrect trials (3.51% of trials) were deleted. Trials in which reaction time was less than 150 ms and greater or less than 3 *SD* from an individual's mean reaction time (1.21% of trials) were eliminated from further analysis.

##### Data of complete shape

**Figure 12.**
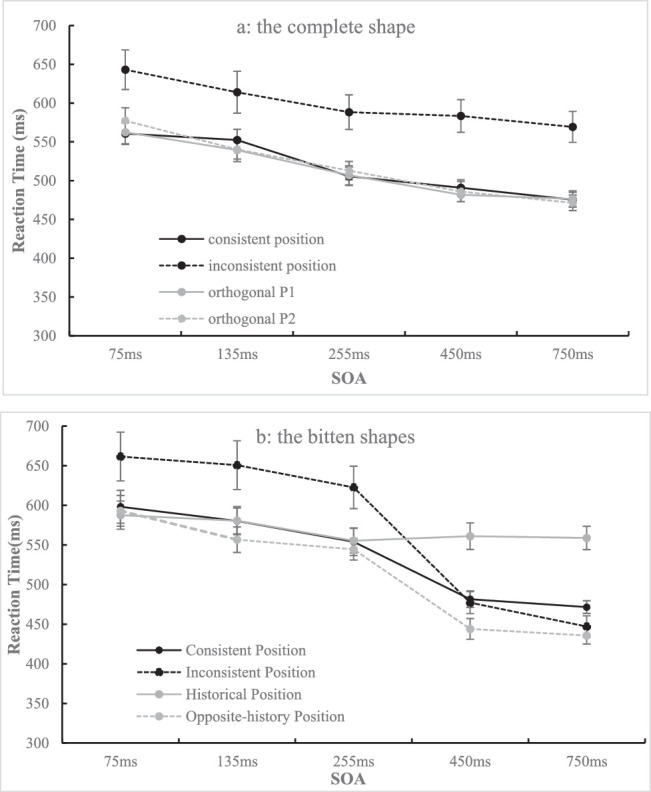
The reaction time under each condition when the target is the complete shape (a) or the bitten shape (b) is shown. The error bar represents standard error.

This part of the data (see Figure 12a) was analyzed in a 4 (position: consistent position, inconsistent position, orthogonal P1, orthogonal P2) × 5 (SOA: 75, 135, 255, 450, 750 ms) repeated-measures ANOVA. The results showed that the main effect of position was significant, *F*(1.46, 35.03) = 17.39, *p* < 0.001, η_p_^2^ = 0.420, and post hoc comparisons with Bonferroni's correction revealed that the inconsistent position (*M* = 599.62 ms, *SD* = 111.38) resulted in a longer RT than the consistent position (*M* = 516.99 ms, *SD* = 46.04, *t*(24) = 4.70, *p* = 0.001, *d_z* = 0.94), orthogonal P1 (*M* = 513.46 ms, *SD* = 51.45, *t*(24) = 4.55, *p* = 0.001, *d_z* = 0.91), and orthogonal P2 (*M* = 517.57 ms, *SD* = 54.59, *t*(24) = 4.35, *p* = 0.001, *d_z* = 0.87). The main effect of the SOA was also significant, *F*(1.53, 36.59) = 37.98, *p* < 0.001, η_p_^2^ = 0.613, and post hoc comparisons with Bonferroni's correction revealed that 75 ms (*M* = 585.88 ms, *SD* = 78.73) resulted in a longer RT than 135 ms (*M* = 547.95 ms, *SD* = 68.18, *t*(24) = 6.09, *p* < 0.001, *d_z* = 1.22), 255 ms (*M* = 528.47 ms, *SD* = 59.66, *t*(24) = 8.27, *p* < 0.001, *d_z* = 1.65), 450 ms (*M* = 510.51 ms, *SD* = 47.57, *t*(24) = 7.45, *p* < 0.001, *d_z* = 1.49), and 750 ms (*M* = 498.15 ms, *SD* = 41.25, *t*(24) = 6.98, *p* < 0.001, *d_z* = 1.40). In addition, 135 ms resulted in a longer RT than 255 ms (*t*(24) = 4.23, *p* < 0.001, *d_z* = 0.85), 450 ms (*t*(24) = 5.11, *p* < 0.001, *d_z* = 1.02), and 750 ms (*t*(24) = 4.97, *p* < 0.001, *d_z* = 0.99), and 255 ms also resulted in a longer RT than 750 ms (*t*(24) = 3.89, *p* = 0.007, *d_z* = 0.78). The interaction of position and SOA did not reach significance, *F*(6.77, 162.65) = 1.49, *p* > 0.17.

##### Data of bitten shape

This part of the data (see Figure 12b) was analyzed in a 4 (position: consistent position, inconsistent position, historical position, opposite-history position) × 5 (SOA: 75, 135, 255, 450, 750 ms) repeated-measures ANOVA. The results showed that the main effect of position was significant, *F*(2.07, 49.62) = 15.34, *p* < 0.001, η_p_^2^ = 0.390, and post hoc comparisons with Bonferroni's correction revealed that the consistent position (*M* = 537.13 ms, *SD* = 55.66) resulted in a shorter RT mean than the inconsistent position (*M* = 571.83 ms, *SD* = 88.88, *t*(24) = 3.42, *p* = 0.013, *d_z* = 0.68) and historical position (*M* = 568.69 ms, *SD* = 76.75, *t*(24) = 3.40, *p* = 0.014, *d_z* = 0.68). The opposite-history position (*M* = 514.79 ms, *SD* = 45.79) resulted in a shorter RT mean than the inconsistent position (*t*(24) = 4.36, *p* = 0.001, *d_z* = 0.86) and historical position (*t*(24) = 5.54, *p* < 0.001, *d_z* = 1.11). The main effect of SOA was also significant, *F*(1.16, 27.81) = 35.24, *p* < 0.001, η_p_^2^ = 0.595, and post hoc comparisons with Bonferroni's correction revealed that 75 ms (*M* = 610.10 ms, *SD* = 102.84) resulted in a longer RT mean than 135 ms (*M* = 592.05 ms, *SD* = 93.91, *t*(24) = 5.09, *p* < 0.001, *d_z* = 1.02), 255 ms (*M* = 569.15 ms, *SD* = 80.24, *t*(24) = 5.96, *p* < 0.001, *d_z* = 1.19), 450 ms (*M* = 490.96 ms, *SD* = 45.05, *t*(24) = 6.41, *p* < 0.001, *d_z* = 1.28), and 750 ms (*M* = 478.28 ms, *SD* = 39.22, *t*(24) = 6.39, *p* < 0.001, *d_z* = 1.28). Additionally, 135 ms resulted in a longer RT mean than 255 ms (*t*(24) = 4.13, *p* = 0.004, *d_z* = 0.83), 450 ms (*t*(24) = 5.84, *p* < 0.001, *d_z* = 1.17), and 750 ms (*t*(24) = 5.94, *p* < 0.001, *d_z* = 1.19), and 255 ms resulted in a longer RT mean than 450 ms (*t*(24) = 5.25, *p* = 0.004, *d_z* = 1.05) and 750 ms (*t*(24) = 5.58, *p* < 0.001, *d_z* = 1.12). These effects were qualified by a significant interaction, *F*(3.34, 80.25) = 15.08, *p* < 0.001, η_p_^2^ = 0.386. Simple analysis revealed that the effects of position were significant when SOAs were 75 ms (*F*(3, 22) = 5.65, *p* = 0.005, η_p_^2^ = 0.435), 135 ms (*F*(3, 22) = 6.84, *p* = 0.002, η_p_^2^ = 0.482), and 255 ms (*F*(3, 22) = 5.59, *p* = 0.005, η_p_^2^ = 0.433). The inconsistent position resulted in a longer RT mean than any of the others (*p* < 0.01 in each case) under the three SOA conditions. The position also had a significant effect on RT when the SOA was 450 ms, *F*(3, 22) = 7.70, *p* = 0.001, η_p_^2^ = 0.513. The difference in RT between the opposite-history position (*M* = 443.98 ms, *SD* = 65.25) and the consistent position (*M* = 481.55 ms, *SD* = 52.07) approached significance, *p* = 0.057. In addition, the historical position (*M* = 561.08 ms, *SD* = 83.67) resulted in a longer RT mean than the consistent position (*M* = 481.55, *SD* = 52.07, *t*(24) = 4.80, *p* < 0.001, *d_z* = 0.96), inconsistent position (*M* = 477.21 ms, *SD* = 69.45, *t*(24) = 4.20, *p* = 0.002, *d_z* = 0.84), and opposite-history position (*M* = 443.98 ms, *SD* = 65.25, *t*(24) = 4.83, *p* < 0.001, *d_z* = 0.97). When the SOA was 750 ms, the effect of position was significant, *F*(3, 22) = 13.44 *p* < 0.001, η_p_^2^ = 0.647. The opposite-history position resulted in a significantly shorter RT (*M* = 435.70 ms, *SD* = 53.44) than the consistent position (*M* = 471.58 ms, *SD* = 39.80, *t*(24) = 4.12, *p* = 0.002, *d_z* = 0.82). In addition, the historical position (*M* = 558.80 ms, *SD* = 73.24) resulted in a significantly longer RT mean than the consistent position (*M* = 471.58 ms, *SD* = 39.80, *t*(24) = 5.53, *p* < 0.001, *d_z* = 1.11), inconsistent position (*M* = 447.03 ms, *SD* = 68.16, *t*(24) = 6.17, *p* < 0.001, *d_z* = 1.23), and opposite-history position (*M* = 435.70 ms, *SD* = 53.44, *t*(24) = 6.26, *p* < 0.001, *d_z* = 1.25).

### Discussion

First, the participants had relatively consistent judgments about the direction of the shapes used in Experiment 2, and where the smaller end of the shape appeared to be heading was the direction of the shape. The smaller end could be judged as the “head” and became the direction of the object. This was also the case in terms of the bitten shapes. The participants could recover the original shape by using the continuation based on local contours and surface structure or global factors such as symmetry (Schmidt, Phillips, & Fleming, 2019), not to mention that they had seen the corresponding complete shapes in the present experiment.

Experiment 2a seemingly found a significant “larger area” bias, that is, our participants were faster to identify probes that appeared at positions close to the larger region of one shape. Such finding was different from the influence of the inherent directionality of the shape on visual attention (Sigurdardottir, Michalak, & Sheinberg, 2014). Nevertheless, after controlling for the expectation effect, the results of Experiment 2b were similar to those of the previous study, displaying an “orientation bias.” Individuals were much faster in identifying the probe that appeared where the shape headed, demonstrating the influence of the directionality of the shape on the distribution of visual attention. Concerning the bitten shapes, the participants’ responses showed an increasing difference from those to the complete shapes as the SOA grew. When the SOA was shorter, the participants’ responses showed the “orientation” bias. With longer SOA, participants were faster to identify probes that appeared at positions close to the area that remained undamaged. The result indicates that past transformations of a shape can, to some degree, influence the allocation of our attention too. When individuals have much time to parse the objects, they can determine the shape features that the objects have and why or how they have these features (Spröte, Schmidt, & Fleming, 2016). Based on the analysis, they could speculate on the past shape of the object and predict the possible changes that could happen to the object in the near future as well. On the other hand, the effect of extrinsic features on visual attention is greater with a longer processing time, perhaps because, when the object is stationary for a long time, it is less urgent to speculate on its possible motion or location in the future. Under such circumstance, predicting the change in the object's shape becomes more important. Due to the uncertainty of future transformation, it is important to pay attention to the remaining undamaged area.

## General discussion

In the present study, we focused on the effects of the shape features imposed by transformations and the shape features constrained by the inherent directionality of the object on the allocation of visual attention. We first concentrated on the regular shapes that do not have apparent original shape features to explore the effect of shape features imposed by causal history on visual attention. Then, we centered on the irregular shapes with apparent directionality to investigate the robustness of the effect of causal history. Based on the analysis, we found that, although the effect of original shape features on attention is built quickly and automatically, it can be disrupted by shape features caused by causal history with increases in processing time.

### Time course of the effect of shape extrinsic features in visual processing

In Experiments 1 and 2, we presented uninformative cues over a range of stimulus onset asynchronies to the participants and asked them to identify a peripheral target. With respect to complete shapes, when the object was directional, the responses were faster at identifying targets that appeared at locations around the “head” of the shape. This effect occurred relatively rapidly. The effect is similar to that of the arrow (Roberts & Humphreys, 2011) and also consistent with the finding that people are more likely to look in the direction of the object being pointing (Clement, O'Donnell, & Brockmole, 2019), demonstrating again that information about intrinsic shape features (inherent directionality in this study) is automatically incorporated into visual orienting. However, when bitten shapes were presented, in the earlier phase of visual processing, participants’ response patterns were similar to those found when the complete shapes were presented. The nonsignificant effect of the position in Experiment 1 and the “directionality” bias in Experiment 2 that were found when SOAs were shorter resembled those found when the complete shapes were presented. Nevertheless, in the later phase of visual processing, extrinsic shape features caused by transformations played a leading role in the distribution of visual attention, displaying the “prospection” bias, which was implied by responses being faster when the targets appeared at positions that were around the remaining intact parts. These results indicate that there might be a very quick mental completion of the bitten shape, finally ending with a representation of the present bitten shape. This completion can be automatic and task-irrelevant. Individuals could represent two types of shapes of the object, for example, the present shape and its possible past shape, and be able to access different shape features depending on the task requirements (Schmidt & Fleming, 2018). The slow time course of the effects of extrinsic shape features found in Experiments 1 and 2 is similar to the delayed time course of action-related objects effects (Roberts & Humphreys, 2011) and grasp-cueing benefits (Fischer, Prinz, & Lotz, 2008). Shape features related to transformation, such as jagged outlines, are usually complex. However, mere complexity cannot account for the slower response to the probe appeared near the transformed region (Experiment 1c). When having more time to process the shape features of objects, individuals could derive different perceptual organizations from bitten shapes and imposed shapes, leading to distinctive response patterns. Therefore, the effect of bitten shapes on visual attention could arise from a higher-level inference of causal history based on local shape features, not just from a lower-level perception of local shape features.

These findings above might also reflect the operation of a two-process model of orienting toward one object with causal history, with relatively short-lived “reflexive” (involuntary) effects overlapping with, but eventually being replaced by, voluntary orienting effects (Friesen, Ristic, & Kingstone, 2004; Tipples, 2008).

### Causal future based on the extrinsic features

Previous studies (Sigurdardottir, Michalak, & Shenberg, 2014) and the results of Experiment 2 found that individuals were much faster at identifying stimuli that appeared at the position where the object faced. The information about one shape's directionality (i.e., arrows; Tipples, 2002, 2008) can draw observers’ attention to the object's future position, demonstrating that direction, which is constrained by the intrinsic shape features, is related to the object's possible motion path. Similarly, the present experimental results show that the extrinsic shape features imposed by causal history can also be incorporated into visuospatial attention. Many studies have verified that observers can robustly distinguish features caused by different generative processes (Phillips & Fleming, 2020; Schmidt & Fleming, 2018). Even we did not watch the actual change in the shape, but we can infer these dynamic changes in the shape from the static shapes of objects. In the present study, the concavity of a shape could provide our visual system with relatively sufficient information to infer the force that has been applied to the object (Spröte & Fleming, 2013) and even the material of the object (e.g., something edible in this study) (Schmidt, 2019). Based on simple heuristic rules, individuals can infer approximate information about the force, but they might not be able to infer the accurate magnitude and the exact direction of the resultant force. The inference of the general information about past transformation, therefore, causes individuals to pay attention to any area where external force might be applied in the future. We can do this perhaps because information about events is generative and abstract (Kim, Effken, & Shaw, 1995). Kim et al. defined events as geometric transformations that produced a variety of styles of change over different objects. In line with this definition, the researchers found that individuals can accurately find the missing parts in the middle or the end developmental state of an object, suggesting that individuals can infer the growth changes and possible future development states according to the overall shape changes. The informational value of a discrete sample of a dynamic event is not restricted to the specific moment at which the sample is taken but goes beyond that moment to specify prospectively the future and retrospectively the past (Kim, Effken, & Shaw, 1995). Similarly, an object with transformations that have been applied to it can also be regarded as an event. Inferences of the generative processes that shape the object not only can be used to figure out how the object has these shape features but also can be used to predict its future developments and changes within the shape. Some researchers believe that shapes can be represented in terms of transformations, even salient landmarks on the object. For example, Schmidt and Fleming (2016) found that observers were adept at identifying correspondences across complex shape-transforming processes by representing positions in space on and around objects relative to salient landmarks of the object itself. This ability could be the basis for the causal history effect, that is, the guiding effect of a shape's extrinsic features on attention, which might be helpful for understanding how individuals track and grasp the changes in objects.

These step-by-step processes could be related to the different tasks of the visual system at different stages. In the early stage, object recognition is required, although it is not necessary to conceptualize the object. Therefore, the representation of the object based on the original shape features is preferred. Some perceptual information about shapes might serve as immediate guides for our initial hunches (Landau & Leyton, 1999). In the later stage, more resources can be allocated to the external shape features, and participants can therefore infer more information about the object, such as its material properties, edibility, and future behaviors (Schmidt & Fleming, 2018). However, whether processing in the first stage must be completed before it passes on to a subsequent stage is not clear.

## Conclusion

With these findings, we conclude that information about shape features caused by causal history will be incorporated into the allocation of visual attention. Transformations that leave traces in the shape of the object can, to some degree, prospectively specify the future of the object. It should be noted that the effect of causal history on the distribution of visual attention is yet restricted to the bite transformation of objects with no more than 15% of the area removed. However, we believe that almost all types of transformations, as long as we can identify them, could provide information about their future developments. It seems reasonable to argue that such abilities might have produced an adaptive advantage across human evolutionary history. This article proves the hypothesis that “shape is time.” The shape of an object can be used as a window into not only its past but also its future.

## Footnotes

1 Although the effect of position was significant when SOA was 75 ms, post hoc multiple comparisons did not reveal significant differences among the four positions. Only a marginally faster RT in identifying the probes that appeared at the opposite-history position compared to the orthogonal P2 was found, *p* = 0.060.
